# Effects of Silymarin on the In Vivo Pharmacokinetics of Simvastatin and Its Active Metabolite in Rats

**DOI:** 10.3390/molecules24091666

**Published:** 2019-04-28

**Authors:** Ying Li, Yin Wu, Ya-Jing Li, Lu Meng, Cong-Yang Ding, Zhan-Jun Dong

**Affiliations:** National Clinical Drug Monitoring Center, Department of Pharmacy, Hebei Province General Center, Shijiazhuang 050051, China; lyyaoda@126.com (Y.L.); wuyin831@126.com (Y.W.); 15369305382@163.com (Y.-J.L.); M18203213683@163.com (L.M.); dingcy1989@126.com (C.-Y.D.)

**Keywords:** herb-drug interaction, silymarin, simvastatin, simvastatin hydroxyacid, pharmacokinetics

## Abstract

Herein, the effect of silymarin pretreatment on the pharmacokinetics of simvastatin in rats was evaluated. To ensure the accuracy of the results, a rapid and sensitive UPLC–MS/MS method was established for simultaneous quantification of simvastatin (SV) and its active metabolite simvastatin acid (SVA). This method was applied for studying the pharmacokinetic interactions in rats after oral co-administration of silymarin (45 mg/kg) and different concentrations of SV. The major pharmacokinetic parameters, including Cmax, tmax, t_1/2_, mean residence time (MRT), elimination rate constant (λz) and area under the concentration-time curve (AUC_0–12h_), were calculated using the non-compartmental model. The results showed that the co-administration of silymarin and SV significantly increased the Cmax and AUC_0–12h_ of SVA compared with SV alone, while there was no significant difference with regards to Tmax and t_1/2_. However, SV pharmacokinetic parameters were not significantly affected by silymarin pretreatment. Therefore, these changes indicated that drug-drug interactions may occur after co-administration of silymarin and SV.

## 1. Introduction

Cardiovascular diseases are the leading cause of death worldwide, with 17.7 million deaths in 2015, which accounted for 31.6% of the global mortality rate [1]. The economic consequences of cardiovascular diseases are enormous due to the combined burden of health-care expenditures and a decline in economic productivity. Hyperlipidemia is reported as an important risk factor associated with cardiovascular diseases. Accordingly, the effective control of hyperlipidemia in patients with a high risk of cardiovascular diseases is a key for preventing, eliminating or minimizing the impact of the disease [2].

Simvastatin (SV) is a potent inhibitor of 3-hydroxy-3-methyl glutaryl coenzyme A (HMG-CoA) reductase, and is a widespread drug used for patients with hyperlipidemia because of its proven efficacy and safety profile [3,4]. However, the use of SV is partly hindered by the fact that it can induce myopathy in SV-treated patients [5,6,7,8]. In addition, since efficient lipid effect requires high SV doses [9], it is essential to develop new models of drug-SV combinations for treating hypercholesterolemia. SV and its metabolite simvastatin acid (SVA) are metabolized by the cytochrome P450 (CYP) 3A and 2C9 isoenzymes and uridine diphosphate-glucuronosyltransferases (UGTs) which are responsible for the metabolism of many clinically useful drugs [10,11,12,13,14,15]. For this reason, the administration of drugs able to interact with the CYP system and UGTs may impact on simvastatin metabolism and pharmacokinetics.

Silymarin, the bioactive constituent of the milk thistle extract, has been applied for centuries for treating diverse disorders [16,17,18,19,20,21]. Previous studies indicated that silymarin regulates the activities of various CYP isozymes [22,23,24] and UGT, especially UGT isoform 1A1 (UGT1A1) [25,26,27,28]. Studies also indicated that silymarin is able to affect the pharmacokinetics of drugs that are equally metabolized by the CYP450 system [29,30,31,32]. Results from previous works indicated that silymarin was able to protect from Adriamycin-induced cardiotoxicity by inhibiting lipid peroxidation and glutathione depletion [33]. Besides, silymarin was known to improve the antioxidant status in blood and liver and affects plasma lipoprotein profile in an experimental model of diet-induced hypertriglyceridemia [34]. Studies of silybin (a component of silymarin) action also revealed its positive effects on lipid and glucose parameters [35]. Therefore, silymarin can be used as a supplement for effective treatment of different etiologies hyperlipidemia. Hypercholesterolemia is a chronic condition often requiring life-long treatment. As such, the widespread use of SV is found to elevate the risk of its exposure to silymarin. In fact, many patients with hypercholesterolemia may seek herbal medicines or dietary supplements to reduce the adverse effects caused by SV, such as elevated liver enzymes. This increases the likelihood of silymarin and simvastatin co-administration. Therefore, documenting this herb-drug interaction could shed light on the clinical drug administration safety. However, the data are limited with regard to the interactions between silymarin and SV.

In drug-drug interaction studies involving SV, several techniques have been developed for accurate determination of SV and SVA [36,37]. However, no technique has been developed for SV and SVA when SV is combined with silymarin. It is necessary to develop an adequate method for pharmacokinetic studies in SV-silymarin combination.

Therefore, the present study aimed to develop a sensitive UPLC–MS/MS method to determine the concentration of SV and SVA and to apply this method for investigating the possible interaction of silymarin with SV through comparing the pharmacokinetic profiles between groups after oral administration in rats.

## 2. Results

### 2.1. Method Optimization

#### 2.1.1. Optimization of Chromatography and Mass Spectrometry Conditions

**Figure 1:**
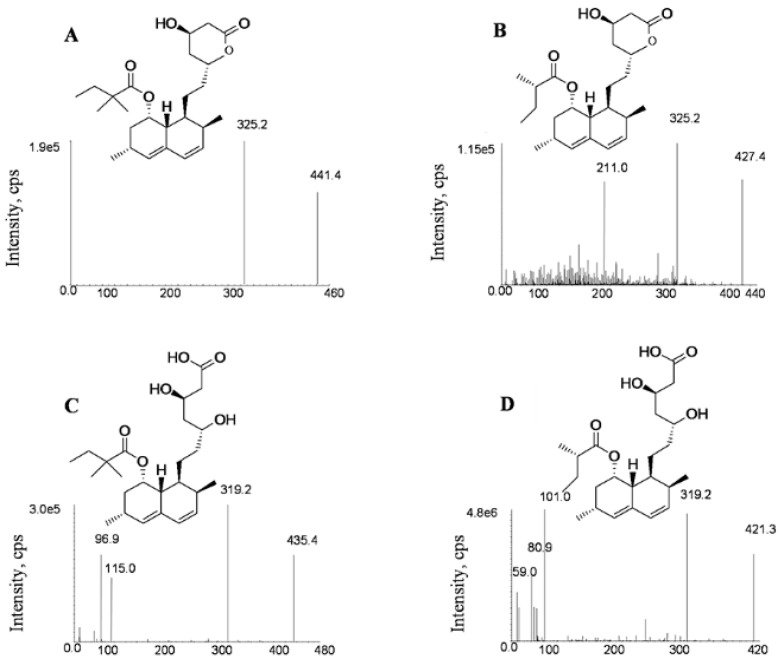
Product ion spectra of simvastatin (**A**), lovastatin (**B**), simvastatin acid (**C**) and lovastatin acid (**D**).

LC–MS/MS conditions were optimized based on previous published works [38] in order to improve the detection sensitivity and run time. Due to the similarities in chemical structure and mass response, both lovastatin (LV)and lovastatin acid (LVA)were chosen as the internal standards (IS) for SV and SVA, respectively (Figure 1).

The electrospray ionization (ESI)ion source was tested in both positive and negative modes to optimize fragmentation conditions. SV and LV were found to show higher responses in the positive mode, while SVA and LVA were more sensitive in the negative mode. The sodium adduct ions [M + Na]^+^ of SV (*m*/*z* 441.4) and LV (*m*/*z* 427.4) were found to provide intense signals in positive mode compared with protonated ions [M + H]^+^. In addition, the [M − H]^−^ of SVA (*m*/*z* 435.4) and LVA (*m*/*z* 421.3) were found to have the highest intensities in the negative mode, which was consistent with previous reports [39,40]. As shown in Figure 1, the precursor for the production of ion transitions used for quantification are *m*/*z* 441.4→325.2 for SV (+), *m*/*z* 427.4→325.2 for LV (+), *m*/*z* 435.4→319.2 (−) for SVA and *m*/*z* 421.3→319.2 (−) for LVA, respectively. The qualitative ion pairs were *m*/*z* 441.4→295.3 for SV (+), *m*/*z* 427.4→295.3 for LV (+), *m*/*z* 435.4→115.2 (−) for SVA and *m*/*z* 421.3→101.1 (−) for LVA, respectively.

In order to achieve the higher responses, mass spectrometer parameters, such as declustering potential (DP) and collision energy (CE) were also optimized. Optimized mass spectrometer parameters are summarized in Table 1.

We also attempted to improve the mobile phase in several trials. The results showed that the LC mobile phase had significant effects on the separation and ionization efficiency of analytes and IS. The use of A: 2.5 mM ammonium acetate B: acetonitrile system as mobile phase was found to produce improved peak shapes and shorter run times when compared to other mobile phase systems. Gradient elution was used for decreasing matrix effects and obtaining a satisfactory resolution. Total run time was found to be within three minutes. The retention times for SV and SVA were 2.38 and 2.10 min, while retention times for the internal standards of LV and LVA were 2.19 and 1.92 min.

#### 2.1.2. Optimization of Plasma Sample Preparation

Three plasma sample preparation techniques, namely protein precipitation, solid phase extraction and liquid–liquid extraction, were evaluated for optimizing the sample preparation. As SV has a high protein binding rate, the protein precipitation technique led to unsatisfactory results with extremely low recoveries. In addition, solid phase extraction was found unsuitable due to the large number of samples required for pharmacokinetic studies. Interestingly, the liquid–liquid extraction method led to satisfactory recoveries, good sensitivity and weak matrix effect. In this method, diverse solvents including n-hexane, ethyl acetate, methylene chloride, tert-butyl methyl ether and different combinations of these solvents showcased that methyl tert-butyl ether led to optimum recovery for analytes and IS. Thus, the liquid–liquid extraction method allowed the obtaining of clean chromatograms and good analytes recovery from plasma samples.

### 2.2. Method Validation

#### 2.2.1. Selectivity

**Figure 2:**
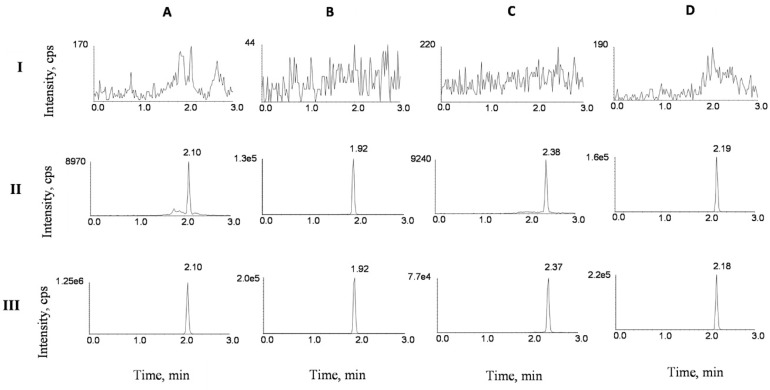
Representative UPLC–MS/MS chromatograms for SVA (**A**), LVA (**B**), SV (**C**) and LV (**D**) in rat plasma samples: (I) a blank plasma sample; (II) a blank plasma sample spiked with SVA, LVA, SV and LV at lower limit of quantitation (LLOQ), and (III) plasma sample of treated rats collected 1 h after the oral administration of a combination of silymarin (45 mg/kg) and SV (80 mg/kg).

Typical UPLC–MS/MS chromatograms SVA, LVA, SV and LV are depicted in Figure 2. No significant peak was obtained from the blank sample. Retention times of SVA, LVA, SV and LV were 2.10, 1.92, 2.38 and 2.19 min in the blank plasma sample spiked with SVA, LVA, SV and LV at lower limit of quantitation (LLOQ), respectively. For the rat plasma samples collected 1 h after the oral administration of a combination of silymarin (45 mg/kg) and SV (80 mg/kg), the retention times of SVA, LVA, SV and LV were 2.10, 1.92, 2.37 and 2.18 min, which was comparable with those obtained in the blank plasma sample spiked with SVA, LVA, SV and LV. No significant interference was found during the retention of SV, SVA and IS.

#### 2.2.2. Linearity and Lower Limit of Quantitation (LLOQ)

The linearity evaluation was performed based on the mean of calibrators studied in three independent batches. The calibration curve for SV and SVA in rat plasma samples showed good linearity within the test concentration range of 0.5–200 ng/mL for SV and 5–2000 ng/mL for SVA (r^2^ > 0.998). Typical linear equations of SVA (1) and SV (2) were:

y = 0.00597x + 0.0673, r^2^ = 0.9981 (1)

y = 0.0299x + 0.0669, r^2^ = 0.9986 (2)

wherein y represents the peak-area ratio of SVA or SV to IS and x stands for the concentrations of SVA and SV.

The deviation of each back-calculated concentration of calibration standard was within ±15% (±20% for LLOQ). This indicated that the calibration curves reliably showed the linear relationship between concentration and the response of analytes. The LLOQ of SVA and SV were 5 and 0.5 ng/mL, respectively.

#### 2.2.3. Accuracy and Precision

Both intra and inter-day precisions were found to be less than 9.3%, and the accuracies of LLOQ and quality control (QC) samples were found to be less than 4.6% for both SVA and SV (Table 2). Therefore, the results were found to be within the acceptable range. These results indicated that the precision, accuracy and reproducibility of the method were sufficient.

#### 2.2.4. Extraction Recovery and Matrix Effect

The average extraction recoveries of SVA and SV respectively ranged from 78.4% to 81.6% and 76.7% to 82.3% for the three different concentrations of QC samples (Table 3). In addition, the mean extraction recovery of LVA and LV was found to be 81.3% and 79.5%. The mean matrix effect values for SVA, SV and the IS were found to be between 92.9% and 97.9% (Table 3). No obvious matrix effects were found for the analytes and IS.

#### 2.2.5. Dilution Integrity

The mean precision (RSD) and accuracy (RE) of SVA and SV were found to be respectively less than 6.0% and ±7.2% after a plasma sample was diluted 10-fold and 20-fold with blank rat plasma (Table 4). The results indicated that when the concentration of SVA or SV was higher than that of the upper limit of quantitation (ULOQ), the plasma samples could be accurately analyzed after a 10-fold and 20-fold dilution.

#### 2.2.6. Stability Studies

Stabilities of SVA and SV in serum samples were analyzed by measuring the concentrations of SV and SVA in the QC samples stored under various storage conditions and comparing the results with nominal values. Assay results indicated that SVA and SV were stable under the storage conditions with an RSD less than 7.0% (Table 5).

### 2.3. Effects of Silymarin on Simvastatin Pharmacokinetics

**Figure 3:**
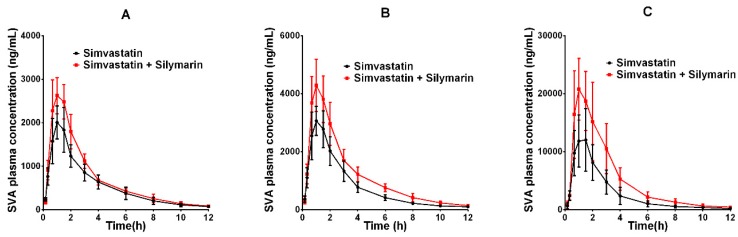
The pharmacokinetic profiles of SVA in rats after oral administration of different doses of SV with or without treatment with silymarin (45 mg/kg). (**A**) Dose of SV at 20 mg/kg; (**B**) dose of SV at 40 mg/kg; (**C**) dose of SV at 80 mg/kg.

**Figure 4:**
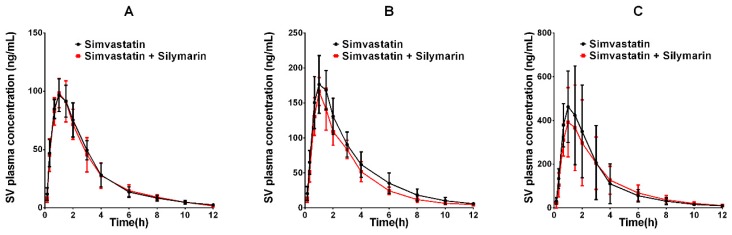
The pharmacokinetic profiles of SV in rats after oral administration of different doses of SV with or without treatment with silymarin (45 mg/kg). (**A**) Dose of SV at 20 mg/kg; (**B**) dose of SV at 40 mg/kg; (**C**) dose of SV at 80 mg/kg.

The developed UPLC–MS/MS method was applied successfully to the in vivo pharmacokinetic studies for the respective groups. The average plasma concentration-time profiles of SVA and SV after oral treatment of SV or co-administration with silymarin and SV are respectively depicted in Figure 3 and Figure 4. The calculated pharmacokinetic parameters are summarized in Table 6. The pharmacokinetic parameters of SVA were significantly different between groups when a single dose of SV was given to silymarin pre-treated rats. The pretreatment of rats with 45 mg/kg silymarin for seven days increased the AUC_0–12h_ of SVA by 1.3-fold (*p* < 0.05) in the low dose (20 mg/kg SV) group, 1.5-fold (*p* < 0.05) in the middle dose (40 mg/kg SV) group, and 1.9-fold (*p* < 0.05) in the high dose (80 mg/kg SV) group. The peak plasma concentrations (Cmax) were also found to increase in various dosage groups. Other parameters such as Tmax and t_1/2_ did not reach statistical significance. Interestingly, no significant differences were observed between groups concerning the pharmacokinetic parameters of SV (Table 6). The pretreatment of rats with 45 mg/kg silymarin for seven days decreased the AUC_0–12h_ of SV by 1.02-fold (*p* > 0.05) in the low dose group, 1.21-fold (*p* > 0.05) in the middle dose group, and 1.04-fold (*p* > 0.05) in the high dose group.

## 3. Discussion

In this study, we have developed an improved UPLC–MS/MS method for studying the effect of silymarin on the pharmacokinetics of SV and its active metabolite SVA in rats. Previous studies have developed UPLC–MS/MS methods for SV and SVA measurement in rat plasma samples and found that the solid phase extraction method was the most appropriate [38]. In the present study, we observed that the liquid–liquid extraction method was the most appropriate. In addition, comparatively to this previous study which found that the total run time was within 4 min [38], we observed that the run time was within 3 min. These results indicated that our method was improved. In the validation process, the linearity and precision were attained, and the results were similar to those previously reported. Moreover, the extraction method used was economical in terms of sampling. Our method was therefore valid for the accurate determination of SV and SVA concentrations and pharmacokinetic studies.

Our pharmacokinetic results revealed that the herb-drug co-administration of SV and silymarin significantly increased SVA plasma concentrations at the three dosage levels, but slightly decreased SV plasma concentration compared to respective single SV administration groups. These results were comparable with findings that Gemfibrozil increases the level of SVA, decreases SV concentration and exerts a minimal effect on SV plasma levels [15]. Previous studies conveyed that the metabolism of SV is complex and involves the oxidative biotransformation mainly by CYP3A [41,42]. Studies also indicated that SV can be hydrolyzed to SVA by esterases, paraoxonases or through chemical approaches [43,44]. UGTs, especially UGT1A1 and UGT1A3, were also found as SV metabolizing proteins which catalyze the formation of glucuronide conjugates of SVA and its reversible conversion to SV [45].

Silymarin has been found to influence on the pharmacokinetics of several drugs [23,30,31]. However, the effect of silymarin on the pharmacokinetics of SV and SVA has not been reported so far. Herein, by applying our improved methods to the detection of SV and SVA, we found that repeated doses of silymarin pretreatment significantly influenced the pharmacokinetics of SVA. Especially, we found that the mean AUC of SVA was increased by 1.3, 1.5 and 1.9-folds in the respective dosage groups. The increased Cmax and AUC of SVA implied that silymarin might inhibit the metabolism of SVA by inhibiting the CYP and/or UTG systems or induce the hydrolysis of SV by regulating esterases or paraoxonases [15]. Despite the increased SVA plasma concentrations, significant differences in SV pharmacokinetics after silymarin consumption were not observed.

CYPs are membrane-bound hemeproteins governing Phase I biotransformation reactions while UGTs are a set of microsomal membrane-bound enzymes involved in Phase II biotransformation. Reactions catalyzed by both CYPs and UGTs induce the attachment of a hydrophilic moiety to xenobiotics to enable their fast elimination from the body. Accumulated data demonstrated in vitro that silymarin inhibits the activities of CYPs and UGTs, especially CYP3A and UGT1A1 [46,47]. The increased concentrations of SVA may be explained by the inhibitory effect of CYP3A and UGT composite activities.

Paraoxonase, an enzyme involved in the hydrolysis of SV [48], may also play a role. Previous studies in dogs indicated that silymarin increases the activity of paraoxonase. The concomitant administration of silymarin with SV might increase the plasma concentrations of SVA and decrease the plasma concentrations of SV. Thus, though it may be speculative, we hypothesized that the effect of silymarin on SV/SVA pharmacokinetics might be equally due induction of paraoxonase activity and inhibition of P-gp. The increase in Cmax and AUC of SVA suggested a possible inhibitory effect on CYPs- and UGTs-mediated SVA metabolism and induction of SV hydrolysis.

Previous studies have shown that silymarin exhibited inhibitory effects on P-gp, an efflux protein. SV is a substrate for P-gp [49]. The concomitant administration of silymarin with SV might increase the plasma concentrations of SV. Indeed, UGT enzymes catalyze the formation of glucuronide conjugates metabolite for excretion of SV and SVA. In addition, the glucuronide conjugates metabolite of SVA could be reversibly converted to SV, which could increase the plasma concentration of SV.

In summary, the results of the pharmacokinetic parameters of SV and SVA may be a combined effect involving CYPs and UGTs and paraoxonases and P-gp. However, further studies are needed to explore the precise mechanism of this phenomenon. This study was conducted in rats rather than humans and since enzymatic differences may exist between species, further clinical studies are still needed.

## 4. Materials and Methods

### 4.1. Reagents and Chemicals

SV and LV were purchased from the National Institute for Food and Drug Control (Beijing, China). SVA and LVA were obtained from Toronto Research Chemicals Inc. (Toronto, ON, Canada). Simvastatin Tablets (Zocor^®^, Merck, Haarlem, The Netherlands) and Silymarin capsules (Legalon^®^, MADAUS GmbH, Cologne, Germany) were bought locally in a pharmacy. HPLC-grade methanol and acetonitrile were purchased from Fisher Scientific (Pittsburgh, PA, USA). A Milli-Q water purification system was obtained from Millipore Corp (Bedford, MA, USA).

### 4.2. UPLC–MS/MS Conditions

Chromatographic analysis was performed using Shimadzu scientific instruments (Shimadzu Corporation; Kyoto, Japan). The UPLC system was interfaced to a mass spectrometer (Thriple Quad 5500 from Applied Biosystems Sciex, Framingham, MA, USA).

The chromatographic separation was carried out on a Waters XBridge BEH C18 column (2.5 × 100 mm, i.d., 2.5 μm, Waters, Ireland) at 40 °C within 3 min. The mobile phase consisted of 2.5 mM ammonium acetate in water (A) and acetonitrile (B). The following gradient condition was used: 0–0.5 min, 55–90% B; 0.5–0.7 min, 90–95% B; 0.7–3 min, 95% B. The flow rate was 0.3 mL/min. The autosampler temperature and injection volume were 4 °C and 3 μL, respectively.

For the detection of SV and LV (IS), the mass spectrometer was set to positive ionization mode, whereas the detection of SVA and LVA (IS) was performed under the negative ionization mode. The multiple reaction monitoring (MRM) mode was used to quantify the analytes and IS. The precursor to product transitions were as follows: SV at 441.4→325.2 (+), LV at 427.4→325.2 (+), SVA at 435.4→319.2 (−) and LVA at 421.3→319.2 (−) (Figure 1). Optimized parameters for MRM mode are listed in Table 1. The detection settings for mass spectrometer were as follows: temperature (TEM), 600 °C; ion spray voltage, 5500 Vin the positive ionization mode and −4500 Vin the negative ionization mode; curtain gas, 20 psi; gas 1, 60 psi and gas 2 (nitrogen), 65 psi.

### 4.3. Preparation of Calibration Standards and Quality Control Samples

The stock solutions of SV, SVA, LV and LVA were obtained by dissolving each reagent in methanol. The concentration of each stock solution was 1000 μg/mL. Working solutions were prepared by diluting the stock solutions with an acetonitrile: water (50:50) mixture. The final concentrations of working solutions ranged from 5–2000 ng/mL for SV and 50–20,000 ng/mL for SVA. The final concentration of the internal control working solution LV was 100 ng/mL while that of LVA was 1000 ng/mL.

Samples for the calibration curve and QC were prepared by mixing 45 μL of blank rat plasma with 5 μL of working solution. The calibration curve was plotted from measurements at concentrations of 0.5, 1, 2, 10, 50, 100 and 200 ng/mL for SV, and 5, 10, 20, 100, 500, 1000 and 2000 ng/mL for SVA. Final concentrations for the QC samples were 1, 10 and 160 ng/mL for SV and 10, 100 and 1600 ng/mL for SVA.

### 4.4. Sample Preparation

Analytes and IS were extracted from plasma samples using a Liquid–liquid extraction (LLE) method. A total of 5 μL IS solution (100 ng/mL LV and 1000 ng/mL LVA) was added to a 50 μL rat plasma, spiked with 10 μL of 0.5 M ammonium acetate buffer (adjusted to pH 4.5 with formic acid) and vortex-mixed for 20 s. After standing for 10 min, 200 μL methyl tert-butyl ether was added, followed by vortex for 2 min. The mixture was then centrifuged at 12,000 rpm for 10 min and the 150 μL upper organic layer transferred to a 1.5 mL centrifuge tube. The pellet was subjected to a second extraction with 100 μL methyl tert-butyl ether, vortex-mixed, centrifuged and the second 100 μL supernatant collected. Supernatants were mixed and dried by evaporation at 40 °C with nitrogen. The residue was reconstituted in 100 μL of initial mobile phase and centrifuged at 12,000 rpm for 5 min. The supernatant was finally collected and 3 μL was used for UPLC–MS/MS analysis.

### 4.5. Bioanalytical Method Validation

Validation of the bioanalytical method was conducted according to the US Food and Drug Administration (FDA)bioanalytical guidelines.

#### 4.5.1. Selectivity

Six independent blank rat plasma samples, blank rat plasma samples spiked with IS and analytes at LLOQ, and actual plasma samples collected from pharmacokinetic studies were analyzed using the LC-MS/MS method. Selectivity was assessed by comparing chromatograms obtained from the six-independent blank rat plasma samples with those obtained from the corresponding spiked plasma.

#### 4.5.2. Linearity and Lower Limit of Quantitation (LLOQ)

Calibration curves were generated by using serial dilutions of SV (0.5 to 200 ng/mL) and SVA (5 to 2000 ng/mL) samples. The linearity of each calibration curve was assessed in three consecutive runs by fitting the peak area ratio (y) of SV or SVA to IS as a function of the nominal concentration (x) of the two analytes. The calibration curves were generated using a 1/x^2^ weighted linear least-squares regression. The acceptable accuracy of the calibration standard was within ±15%, while that of the LLOQ was within ±20%. The LLOQ was the lowest concentration on the calibration curve with the signal-to-noise (S/N) ratio of at least 10:1.

#### 4.5.3. Accuracy and Precision

For determining the intra-day accuracy and precision, five replicates of LLOQ and QC samples from the same day were analyzed. The analysis was performed for three consecutive days. Precision was measured as the relative standard deviation (RSD %) while the accuracy was calculated as the relative error (RE %), which referred to the deviation of the mean measured concentration to the nominal concentration. Both accuracy and precision were within 15%, except for LLOQ for which these variables were within 20%.

#### 4.5.4. Extraction Recovery and Matrix Effect

The extraction recovery of analytes and IS was assessed by computing the average of the response of each concentration and dividing the extracted sample mean by the unextracted (spiked blank plasma extract) sample mean of the corresponding concentration. Comparison with the unextracted samples, and spiked plasma residues, was carried out in order to eliminate matrix effects, giving the true recovery. This procedure was repeated for the three concentrations. The matrix effects were examined by comparing the results of reference standard solutions with and without matrix (the post-extracted blank rat plasma).

#### 4.5.5. Dilution Integrity

A dilution integrity test was conducted to ensure the integrity of analytes in plasma samples with concentrations above the ULOQ. The five replicate samples had a concentration of about five times that of the ULOQ concentration, and were diluted 10- and 20-fold using blank rat plasmas samples. The diluted samples were analyzed using standard calibration curves. The accuracies and precisions of less than 15% were acceptable for the diluted samples.

#### 4.5.6. Stability Studies

Stability studies were designed according to actual experimental conditions the harvested samples would likely experience. Stability was evaluated by examining two concentrations of QC samples (Low-quality controland High-quality control) with five replicates under the following four storage conditions: (i) 2 h at room temperature for short-term stability; (ii) 7 days at −80 °C for long-term stability; (iii) three freeze–thaw cycles between −80 °C and room temperature for freeze–thaw stability; (iv) 24 h in an autosampler at 4 °C for post-preparation stability.

### 4.6. Herb–Drug Interaction Studies

#### 4.6.1. Animals

SV was previously reported to be mainly metabolized by CYP3A4 in humans, CYP3A in female rats and CYP2C11 in male rats [15,41]. The drug interactions in CYP3A-mediated SV metabolism in female rats were found to reflect those in humans [50]. Therefore, female rats were used in the present study.

Female Sprague-Dawley rats (220–280 g) were purchased and housed in the Laboratory Animal Center of Hebei Medical University. The animal protocols were approved by the Animal Center of Hebei Medical University under the approval number 201905. All experimental procedures were strictly conducted following the Guidance for the Care and Use of Laboratory Animals of the US National Institute of Health. Animal were maintained in a temperature controlled (25 ± 2 °C) room at the humidity rate of 60–70% with dark/light cycle of 12 h/12 h. All animals had free access to food and water. The rats were acclimatized for seven days to laboratory conditions before starting the experiments.

#### 4.6.2. Drug Administration

Thirty-six rats were randomly divided into six groups of six rats in each: low dose (20 mg/kg), middle dose (40 mg/kg) and high dose (80 mg/kg) simvastatin groups and their corresponding controls. All rats were administered 0.5% CMC-Na (control group for different corresponding SV doses) or silymarin (45 mg/kg, co-administration group) daily by gastrogavage for six days. Before the seventh administration, the rats were fasted overnight with access only to water. Thirty minutes after the seventh treatment, 20, 40 and 80 mg/kg SV doses were respectively administered to the corresponding control or co-administration rats by gastrogavage.

Both silymarin and SV were dissolved in 0.5% sodium carboxymethyl cellulose (CMC-Na) to form a fine suspension because of their poor solubility. The silymarin doses were chosen by conversion from a human clinical dose to an animal dose under FDA guidance [51] using the following formula:

(3) $$HED\;\left(mg/kg\right)=Animal\;Dose\;\left(mg/kg\right)\;\times {\left(\frac{Animal\;Weight\;\left(kg\right)}{Human\;Weight\;\left(kg\right)}\right)}^{0.33}$$

where: *HED* = Human equivalent dose (mg/kg).

#### 4.6.3. Blood Sampling

Blood samples (0.2 mL) were drawn from each rat at 0.167, 0.33, 0.67, 1, 1.5, 2, 3, 4, 6, 8, 10 and 12 h after SV administration, transferred to heparin tubes and centrifuged at 4000 rpm for 10 min. Plasma was separated from the blood and stored at −80 °C pending further analysis.

#### 4.6.4. Analysis of Pharmacokinetic Data

Non-compartmental analysis was performed to calculate the pharmacokinetic parameters using Phoenix WinNonlin™ 7.0 (Pharsight Corporation, Sunnyvale, CA, USA). Parameters analyzed included the area under the concentration-time curve (AUC_0–12h_) and that extrapolated to infinity (AUC_0-∞_), area under the moment curve (AUMC), maximum observed plasma concentration (Cmax), time of Cmax (Tmax), total half-life (t_1/2_), mean residence time (MRT), elimination rate constant (λz) and total body clearance (CL). The Cmax and Tmax were directly deduced from the plasma concentration–time curve. The elimination rate constant (λz) was calculated from points of the terminal phase of the semi-log regression of the concentration–time curve. The elimination half-life (t_1/2_), which is defined as the time taken for plasma concentration of a given drug to decrease by 50% of its initial dose, was calculated as 0.693/λz. The area under curve from time 0 to infinity (AUC_0-∞_) was estimated as AUC_0-t_ + Ct/λz, where Ct is the plasma concentration of the last measurable sample and AUC_0-t_ was calculated using the linear trapezoidal rule. CL was computed as dose/AUC_0-∞_.

### 4.7. Statistical Analysis

Unless otherwise noted, all results were expressed as the mean ± standard deviation (SD). Statistical analysis was performed using the GraphPad prism software. Student’s *t*-test was used for inter-group comparison between the SV alone and co-administration groups, with normally distributed variables. Finally, a Mann–Whitney test was used for those with skewed variables. A *p*-value of *p* < 0.05 was considered statistically significant.

## 5. Conclusions

In this study, a sensitive and selective UPLC–MS/MS method to quantify SV and SVA in rat plasma samples was developed, validated and subsequently applied for assessing the effect of silymarin on the pharmacokinetic effect of SV at different dosage levels in rats. According to the results of the present study, silymarin and SV co-administration was found to appreciably increase the Cmax and AUC_0–12h_ of SVA, while no significant difference in Tmax and t_1/2_ was found when compared to the SV group alone. However, silymarin was not found to significantly affect SV pharmacokinetic parameters. These results might be a combined effect involving CYPs and UGTs and paraoxonases and P-gp. Consequently, as the plasma concentration of SVA was found to increase when SV was co-administered with silymarin, the possibility of pharmacokinetic interactions between silymarin and SV should be considered to avoid potential adverse effects.

## Figures and Tables

**Table 1:** Experimental setting for the tandem mass-spectrometer for the analysis of simvastatin (SV), simvastatin acid (SVA), LV (IS) and LVA (IS).

Experimental Setting	SV	LV	SVA	LVA
Quantifier transition	441.4→325.2	427.4→325.2	435.4→319.2	421.3→319.2
Qualifier transition	441.4→295.3	427.4→295.3	435.4→115.2	421.3→101.1
Declustering potential (DP), V	130	130	−100	−100
Collision energy (CE), V	32	30	−25	−25
Collision cell exit potential (CXP), V	10	10	−10	−10
Entrance potential (EP), V	14	14	−17	−17

**Table 2:** Precision and accuracy of SVA and SV in rat plasma (mean ± S.D, *n* = 5).

Analytes	Spiked Con. (ng/mL)	Intra-Day (*n* = 5)			Inter-Day (*n* = 15)		
		Measured con. (ng/mL)	Precision (RSD %)	Accuracy (RE %)	Measured con. (ng/mL)	Precision (RSD %)	Accuracy (RE %)
SVA	5.0	5.23 ± 0.36	6.9	4.6	5.11 ± 0.32	6.3	2.2
	10.0	9.72 ± 0.38	3.9	−2.9	9.71 ± 0.38	4.0	−2.9
	100.0	99.8 ± 4.13	4.1	−0.2	98.79 ± 3.42	3.5	−1.2
	1600.0	1644 ± 70.77	4.3	2.8	1642 ± 75.42	4.6	2.6
	0.5	0.52 ± 0.05	9.3	3.2	0.508 ± 0.04	8.2	1.7
SV	1.0	1.00 ± 0.04	3.5	−0.9	0.992 ± 0.03	3.1	−0.8
	10.0	9.84 ± 0.55	5.6	−1.6	9.83 ± 0.41	4.1	−1.7
	160	157.2 ± 6.83	4.4	−1.7	155.2 ± 6.62	4.3	−3.0

**Table 3:** Extraction recoveries and matrix effects of SVA, SV and IS (LVA and LV) in rat plasma (mean ± S.D, *n* = 5).

Analytes	Spiked con. (ng/mL)	Extraction Recovery (%)	Matrix Effect (%)
SVA	10	78.4 ± 3.9	92.9 ± 3.0
	100	81.6 ± 3.9	93.9 ± 5.9
	1600	80.1 ± 2.7	97.7 ± 4.8
SV	1.0	76.7 ± 4.4	92.6 ± 4.8
	10	82.3 ± 3.2	97.8 ± 1.6
	160	81.5 ± 2.1	93.3 ± 3.0
LVA	100	81.3 ± 3.7	95.5 ± 1.8
LV	10	79.5 ± 4.2	97.9 ± 2.2

**Table 4:** Dilution integrity of SVA and SV in rat plasma (mean ± SD, *n* = 5).

Analytes	Spiked con. (ng/mL)	Dilution Factor	Measured con. (ng/mL)	Precision (RSD %)	Accuracy (RE %)
SVA	10,000	10	9491.8 ± 451.1	4.8	−5.1
	10,000	20	9276.2 ± 484.5	5.2	−7.2
SV	1000	10	947.0 ± 44.4	4.7	−5.3
	1000	20	956.5 ± 57.4	6.0	−4.3

**Table 5:** Stability of SVA and SV in rat plasma under various storage conditions (mean ± SD, *n* = 5).

Analytes	Storage Conditions	Spiked con. (ng/mL)	Measured con. (ng/mL)	Precision (RSD %)	Accuracy (RE %)
SVA	Short-term stability (25 °C, 2 h)	10	9.83 ± 0.64	6.5	−1.7
		1600	1629 ± 60.22	3.7	1.9
	Long-term stability (−80 °C, 7 days)	10	9.91 ± 0.63	6.3	−0.9
		1600	1608 ± 33.31	2.1	0.6
	Freeze–thaws stability (−80 to 25 °C)	10	9.95 ± 0.40	4.0	−0.5
		160	1623 ± 79.81	4.9	1.5
	Post-preparation stability (4 °C, 24 h)	10	9.91 ± 0.50	5.1	−0.9
		160	1663 ± 68.78	4.1	3.9
SV	Short-term stability (25 °C, 2 h)	1.0	0.956 ± 0.05	5.4	−4.4
		160	155.9 ± 8.15	5.2	−2.6
	Long-term stability (−80 °C, 7 days)	1.0	1.008 ± 0.05	4.8	0.8
		160	159.0 ± 11.10	7.0	−0.6
	Freeze–thaws stability (−80 °C to 25 °C)	1.0	0.977 ± 0.03	3.3	−2.3
		160	155.5 ± 10.96	7.0	−2.8
	Post-preparation stability (4 °C, 24 h)	1.0	0.972 ± 0.05	5.6	−2.8
		160	156.2 ± 6.63	4.2	−2.3

**Table 6:** The pharmacokinetic parameters of SVA and SV in rats after oral administration of different doses of SV (20, 40 and 80 mg/kg, *n* = 6) with or without silymarin.

	Low Dose (20 mg/kg)		Middle Dose (40 mg/kg)		High Dose (80 mg/kg)	
PK Parameter (Unit)	with Silymarin	without Silymarin	with Silymarin	without Silymarin	with Silymarin	without Silymarin
**SV**						
AUC_0–12h_ (ng*h/mL)	327.0 ± 66.7	332.2 ± 48.5	530.3 ± 70.6	641.5 ± 117.2	1357.4 ± 635.2	1409.0 ± 736.6
AUC_0-∞_ (ng*h/mL)	332.7 ± 67.0	340.8 ± 50.7	541.4 ± 72.2	658.3 ± 124.2	1399.3 ± 632.0	1442.6 ± 740.5
AUMC (h^2^*ng/mL)	982.5 ± 240.8	984.6 ± 224.6	1571.2 ± 280.8	2061.9 ± 600.9	4277.2 ± 1884.4	3929.8 ± 2214.5
**Cmax (ng/mL)**	101.4 ± 11.8	104.4 ± 9.3	179.1 ± 10.5	203.1 ± 24.9	435.7 ± 171.8	482.8 ± 168.8
**Tmax (h) ^a^**	1.0(0.67,1.12)	1.0(0.67,1.5)	1.0(0.92,1.12)	1.0(0.67,1.5)	1.0(0.92,1.5)	1.0(0.92,1.12)
**t_1/2_ (h)**	2.0 ± 0.2	2.3 ± 0.4	2.1 ± 0.2	2.1 ± 0.2	2.4 ± 0.7	2.4 ± 0.4
**MRT (h)**	3.0 ± 0.2	2.9 ± 0.3	3.0 ± 0.2	3.2 ± 0.4	3.2 ± 0.3	2.7 ± 0.4
**CL (L/h/kg)**	62.1 ± 11.0	59.7 ± 8.1	74.6 ± 9.2	62.8 ± 14.1	66.1 ± 25.5	69.8 ± 37.4
**λz (1/h)**	0.35 ± 0.03	0.31 ± 0.05	0.32 ± 0.02	0.32 ± 0.02	0.30 ± 0.09	0.30 ± 0.05
**SVA**						
AUC_0–12h_ (ng*h/mL)	8559.1 ± 831.1 *	6701.5 ± 1275.3	13,977.7 ± 2522.7 *	9477.1 ± 1786.7	62,658.6 ± 15,666.7 *	33,376.9 ± 13,560.7
AUC_0-∞_ (ng*h/mL)	8797.4 ± 872.2 *	6933.3 ± 1315.8	14,445.3 ± 2600.0 *	9776.4 ± 1785.3	63,887.5 ± 16,153.3 *	34,015.5 ± 13,875.1
AUMC (h^2^*ng/mL)	26,604.5 ± 3472.7	21,744.3 ± 5392.1	44,543.6 ± 8982.8 *	27,939.8 ± 5737.6	177,142.2 ± 54,541.3 *	87,850.4 ± 41,089.1
**Cmax (ng/mL)**	2795.5 ± 495.0 *	2255.0 ± 266.0	4623.3 ± 690.4 *	3276.7 ± 573.0	21,833.3 ± 5689.9 *	12,936.7 ± 5313.4
**Tmax (h) ^a^**	1.25(0.92,1.5)	1.0(0.67,1.5)	1.0(0.67,1.1)	1.0(0.67,1.5)	1.0(0.92,1.63)	1.25(0.92,1.5)
**t_1/2_ (h)**	2.2 ± 0.2	2.4 ± 0.4	2.4 ± 0.3	2.5 ± 0.4	2.1 ± 0.4	2.3 ± 0.3
**MRT (h)**	3.1 ± 0.3	3.2 ± 0.2	3.2 ± 0.2	3.0 ± 0.2	2.8 ± 0.4	2.6 ± 0.2
**λz (1/h)**	0.32 ± 0.02	0.29 ± 0.05	0.29 ± 0.03	0.28 ± 0.04	0.33 ± 0.07	0.3 ± 0.04

AUC: area under the plasma concentration-time curve; Cmax: maximum plasma concentration; Tmax: time to Cmax; t1/2: terminal elimination half-life. ^a^: Tmax (h) was expressed as Median (range). * *p* < 0.05.
